# Spatial-temporal characteristics and the epidemiology of haemorrhagic fever with renal syndrome from 2007 to 2016 in Zhejiang Province, China

**DOI:** 10.1038/s41598-018-28610-8

**Published:** 2018-07-06

**Authors:** Haocheng Wu, XinYi Wang, Ming Xue, Chen Wu, Qinbao Lu, Zheyuan Ding, Yujia Zhai, Junfen Lin

**Affiliations:** 1 Zhejiang Province Center for Disease Control and Prevention, Hangzhou, Zhejiang Province China; 2 Hangzhou Centre for Disease Control and Prevention, Hangzhou, Zhejiang Province China; 3 Key Laboratory for Vaccine, Prevention and Control of Infectious Disease of Zhejiang Province, Hangzhou, Zhejiang Province China

## Abstract

Zhejiang Province is one of the six provinces in China that has the highest incidence of haemorrhagic fever with renal syndrome (HFRS). Data on HFRS cases in Zhejiang Province from January 2007 to July 2017 were obtained from the China Information Network System of Disease Prevention and Control. Joinpoint regression analysis was used to observe the trend of the incidence rate of HFRS. The monthly incidence rate was predicted by autoregressive integrated moving average(ARIMA) models. Spatial autocorrelation analysis was performed to detect geographic clusters. A multivariate time series model was employed to analyze heterogeneous transmission of HFRS. There were a total of 4,836 HFRS cases, with 15 fatal cases reported in Zhejiang Province, China in the last decade. Results show that the mean absolute percentage error (MAPE) of the modelling performance and the forecasting performance of the ARIMA model were 27.53% and 16.29%, respectively. Male farmers and middle-aged patients account for the majority of the patient population. There were 54 high-high clusters and 1 high-low cluster identified at the county level. The random effect variance of the autoregressive component is 0.33; the spatio-temporal component is 1.30; and the endemic component is 2.45. According to the results, there was obvious spatial heterogeneity in the endemic component and spatio-temporal component but little spatial heterogeneity in the autoregressive component. A significant decreasing trend in the incidence rate was identified, and obvious clusters were discovered. Spatial heterogeneity in the factors driving HFRS transmission was discovered, which suggested that a targeted preventive effort should be considered in different districts based on their own main factors that contribute to the epidemics.

## Introduction

Hantaviruses represent an emerging global threat to public health, affecting approximately 30,000 humans annually, which may lead to hantavirus pulmonary syndrome (HPS) in the Americas and haemorrhagic fever with renal syndrome (HFRS) in Europe and Asia^1^. HFRS is an important rodent-borne disease characterized by fever, haemorrhage, kidney disease, headache, back pain, abdominal pain and hypotension^2–4^. Severe HFRS can develop into five phases: the febrile, hypotensive (shock), oliguric, diuretic and convalescent phases^5^. Human beings are infected by hantaviruses through the inhalation of aerosolized excreta (including faeces, urine and saliva) from infected animals^1,6^. Involvement in outdoor work, such as rural- and forest-related activities, a peridomestic rodent presence, exposure to potentially infected dust and outdoor military training are usually risk factors for human infection with these viruses^1^. According to previous studies^3,4,7–9^, more than 10,000 cases are reported annually in China, representing almost 90% of all cases worldwide. This disease remains a severe public health issue in mainland China^10^. The top five provinces with the highest HFRS incidence are Heilongjiang, Shaanxi, Shandong, Liaoning and Jilin, which are all located in northern China. Zhejiang, ranked sixth highest in HFRS incidence, is located in the Yangtze River Delta region of the southeast China^4^. The objective of this study is to identify the epidemiological characteristics of HFRS in Zhejiang from 2007 to 2016. The joinpoint regression analysis was used to observe the trend in HFRS incidence. Autoregressive integrated moving average (ARIMA) models were applied to predict the incidence of HFRS. The geographic clusters were detected by the spatial autocorrelation analysis. We used multivariate time series model to analyze the heterogeneous transmission of this disease. These models additively decompose HFRS risk into endemic, autoregressive and spatiotemporal components.

## Materials and Methods

### Ethical review

This study was reviewed and approved by the Ethics Committee of the Zhejiang Provincial Center for Disease Control and Prevention. All the data of the individuals were kept confidential as requested. Verbal informed consent was obtained from all the patients before diagnosing and reporting to the China Information Network System of Disease Prevention and Control. All the methods employed in the study were in accordance with the applicable guidelines and regulations.

### Profile of zhejiang province

**Figure 1:**
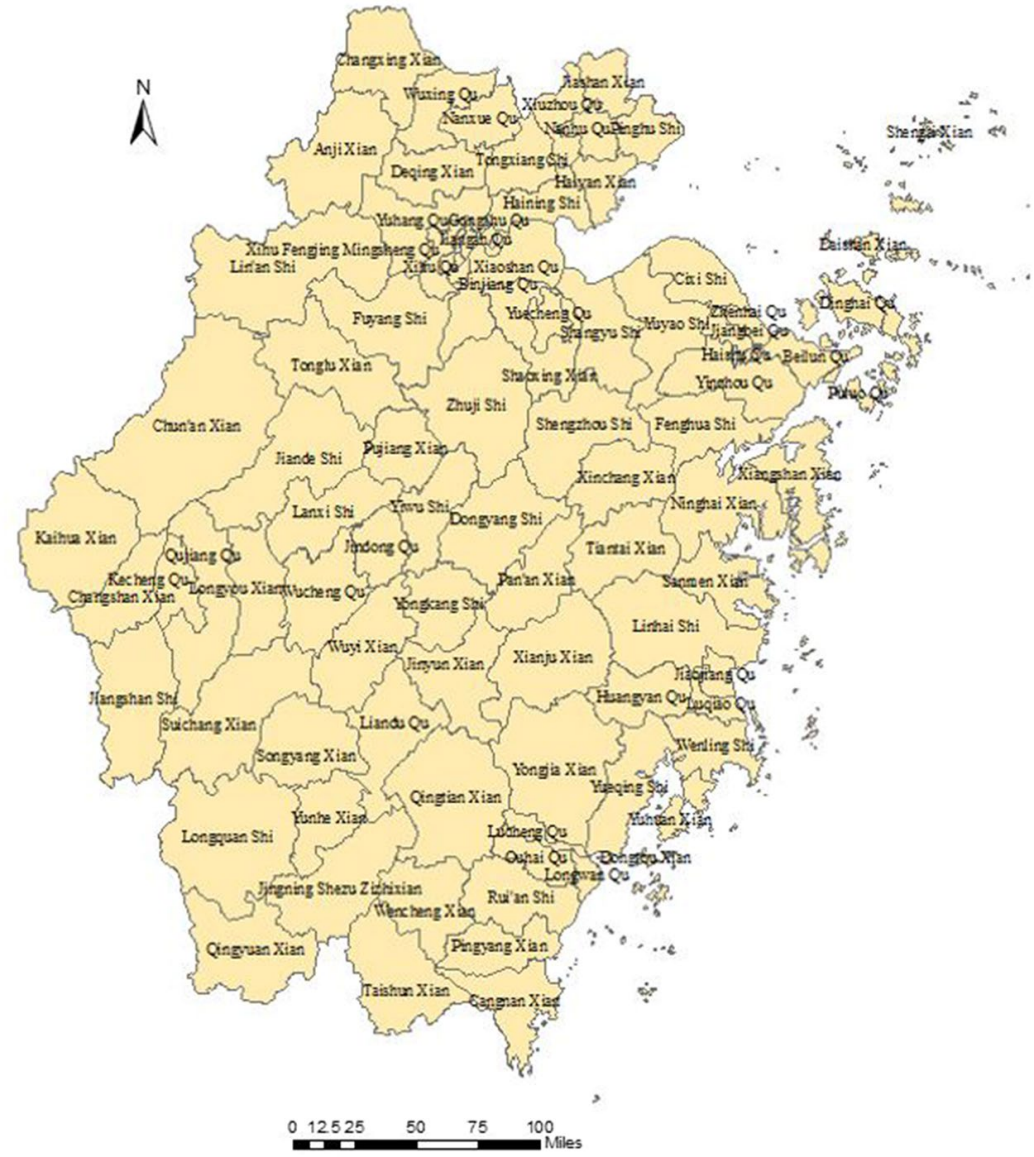
Maps of Zhejiang Province, China with area names. This map was created by ArcGIS software (version 10.1, ESRI Inc.; Redlands, CA, USA). The homepage for the ArcGIS software was https://www.esri.com/.

Zhejiang Province is located in southeast China between longitudes 118°E-123°E and latitudes 27°N-32°N. There are two sub-provincial cities (Hangzhou and Ningbo) and nine prefecture-level cities including Wenzhou, Huzhou, Jiaxing, Shaoxing, Jinhua, Zhoushan, Quzhou, Taizhou and Lishui, which cover 90 counties (Fig. 1).

### The data collection

Any human HFRS case diagnosed in the hospital must be reported through the China Information Network System of Disease Prevention and Control by the medical staff. The data of the HFRS cases in Zhejiang Province from January 2007 to July 2017 and the data of the populations at the county level were obtained from this network system. Individual information on cases and deaths were imported, and surveillance information, including demographic characteristics and geographic and temporal distributions, were computed by the system. The definition of reporting cases referred to the ‘Diagnostic criteria for epidemic haemorrhagic fever’ (WS 278–2008) of China.

### Joinpoint regression

Joinpoint regression is a model used to describe continuous changes in the incidence trend., The grid-search method was used to fit the regression function, with unknown joinpoints assuming constant variance and uncorrelated errors^11^. The joinpoint regression model for the observations $({x}_{1},{y}_{1}),\mathrm{...},({x}_{n},{y}_{n})$, $({x}_{1}\le \mathrm{...}\le {x}_{n})$ without a loss of generality is written as

1 $$E[y/x]={\beta }_{0}+{\beta }_{1}+{\delta }_{1}{(x-{\tau }_{1})}^{+}+\mathrm{...}+{\delta }_{k}{(x-{\tau }_{k})}^{+},$$

where y is the outcome of interest, x is the time variable, the ${\tau }_{k}\mbox{'}s$ are the unknown joinpoints, and ${\alpha }^{+}=\alpha$ for $\alpha$ > 0 and 0 otherwise.

The approximate permutation test was used to find the number of significant joinpoints, each p-value was obtained using Monte Carlo methods, and the overall asymptotic significance level was maintained through a Bonferroni correction. The objective indicator was the annual percent changes (APCs) of each period segment, which was estimated according to the following formula:

2 $$APCi=[(Exp({\beta }_{i})-1]\times 100,$$

where ${\beta }_{i}$ represents the slope of the period segment^11,12^.

### Time-series estimation

The ARIMA model and the decomposed models have been widely applied to predict trends in many infectious disease incidences^13–16^. The formula of the ARIMA model is written as:

3 $${W}_{t}=\mu +\frac{\theta (B)}{\varphi (B)}{a}_{t},$$

where ${W}_{t}$ is the response series ${Y}_{t}$, or a difference of the response series. $\mu$ is the intercept; $B$ is the backshift operator($B{X}_{t}={X}_{t-1}$).

$\varphi (B)$ is the autoregressive operator, represented as a polynomial in the backshift operator:

4 $$\varphi (B)=1-{\varphi }_{1}B-\mathrm{...}-{\varphi }_{p}{B}^{p}$$

where *p* is the order of the autoregressive process; $\varphi$ is the autoregressive coefficient;

$\theta (B)$ is the moving-average operator, represented as a polynomial in the backshift operator:

5 $$\theta (B)=1-{\theta }_{1}B-\mathrm{...}-{\theta }_{q}{B}^{q}$$

where *q* is the order of the moving-average process; $\theta$ is the moving-average coefficient; and ${a}_{t}$ is the random error.

The formula for simple (nonseasonal) differencing is written

6 $${W}_{t}={(1-B)}^{d}{Y}_{t}$$

The formula for seasonal differencing is

7 $${W}_{t}={(1-B)}^{d}{(1-{B}^{s})}^{D}{Y}_{t}$$

where $d$ is the degree of nonseasonal differencing; $D$ is the degree of seasonal differencing, and the length of the seasonal period is represented by *s*, which is 12 for monthly series. The formula “(3)” suggests that the model predicts a value as a linear combination of its own past value and past errors. The nonseasonal model is usually represented by ARIM A (*p*, *d*, *q*), and the seasonal model is normally represented by ARIM A (*p*, *d*, *q*) × (*P*, *D*, *Q*)_s_. The term (*p*, *d*, *q*) gives the order of the nonseasonal part, and (*P*, *D*, *Q*)_s_ gives the order of the seasonal part.

The ARIMA analysis is usually divided into three stages including the identification stage, the estimation and diagnostic stage and the forecasting stage.

In the first stage, the white noise test was done to assess if there is any information in the series to model. This is an approximate statistical test of the null hypothesis, in which none of the autocorrelations of the series is up to a given lag. If this is true for all lags, there will be no information in the series to model. If the hypothesis is rejected, the next step of stationarity tests is performed to determine if differencing is needed, sincethe stationarity of the series is necessary for constructing the model. An easy way to assess nonstationarity is to visually examine a graph of the series over time to see if it has a trend, seasonality, or other nonstationary patterns. Another way is to do the stationarity test, withthe null hypothesis being the time series is nonstationary.If the series is not stationary, an appropriate difference should be calculated to make the series stationary before moving onto the next step. In the second stage, the candidate models were identified according to the autocorrelations and the partial autocorrelations. The nonseasonal autoregressive order (*p*) and the seasonal autoregressive order (*P*) were determined using partial autocorrelation functions (PACF); the nonseasonal moving average order (*q*) and the seasonal moving average order (*Q*) were determined by the autocorrelation functions (ACF). The estimation of the coefficient including $\varphi$ and $\theta$ was calculated by the conditional least squares method. In the last stage, the adequacy of the established model for the series was verified by employing white noise tests. The tests examined whether the residuals were independent and normally distributed, indicating that the residual series contains any additional information that might be used by a more complex model^17,18^. The optimum model was selected according to the Akaike’s information criterion (AIC) and the Schwartz Bayesian criterion (SBC). Smaller AIC and SBC values represent a superior model^17^. In addition, the mean absolute percentage error (MAPE) was calculated to check the accuracy of the model and to compare the results with that of other studies. Once the model was adopted, we forecasted the short-term trend of the monthly incidence.

### Spatial autocorrelation analysis

Spatial analysis was applied to describe geographic variation and to detect the clustering regions^19^. A global and local autocorrelation analysis using the Moran’s I was carried out to identify spatial autocorrelations. Global autocorrelation demonstrates the spatial distribution clusters over areas^20^. The Local Moran’s I(LMi) Index was used to classify the autocorrelations as positive and negative values. When there were similar high values or low values of the incidence rates, the locations were defined as having a positive autocorrelation (represented as high-high or low-low autocorrelation). If opposite high and low values occurred, they were considered to have a negative autocorrelation (represented as high-low or low-high autocorrelation)^21^.

### Multivariate time series model

The multivariate time series model established by Held and Paul was designed for spatially and temporally aggregated surveillance data. The $${Y}_{i,t}$$ is the disease count in region $$i=1,\mathrm{...},$$$$I$$ at time $$t=1,\mathrm{...},T$$. The count $${Y}_{i,t}$$ is formally assumed to follow a negative binomial distribution $${Y}_{it}/{Y}_{i,t-1} \sim NegBin\,({\mu }_{it},\psi )$$ $$i=1,\mathrm{...},I,t=1,\mathrm{...},T$$, with an additively decomposed mean

8 $${\mu }_{it}={\nu }_{it}{e}_{it}+{\lambda }_{it}{Y}_{i,t-1}+{\varphi }_{it}\sum _{j\ne i}{\omega }_{ji}{Y}_{j,t-1}$$

where $\psi$ is an overdispersion parameter such that the conditional variance of ${Y}_{it}$ is ${\mu }_{it}(1+{\rm{\Psi }}{\mu }_{it})$. The Poisson distribution results in a special case if $\psi$ = 0. The first component ${\nu }_{it}{e}_{it}$ represents the endemic component, which captures exogenous factors such as population, socio-demographic variables, long-term trends, seasonality, and theclimate. The endemic mean is proportional to an offset of known expected counts ${e}_{it}$, typically reflecting the population at risk. Here, the population at the county level as a multiplicative offset was incorporated into the endemic component. The next two components are observation-driven epidemic components. The second component ${\lambda }_{it}{Y}_{i,t-1}$ is an autoregression on the number of cases at the previous time point. The third component ${\varphi }_{it}\,\sum _{j\ne i}{\omega }_{ji}\,{Y}_{j,t-1}$ denotes the spatio-temporal characteristics capturing the transmission from the other units. Each parameter ${\nu }_{it}$, ${\lambda }_{it}$, and ${\varphi }_{it}$ is a linear predictor of the form

9 $$\mathrm{log}(\cdot it)={\alpha }^{(\cdot )}+{b}_{i}^{(\cdot )}+{\beta }^{{(\cdot )}^{T}}{z}_{it}^{(\cdot )},$$

where “.” is one of $\nu ,\lambda ,\varphi$; ${\alpha }^{(.)}$ are intercepts; ${b}_{i}^{(\cdot )}$ denotes the random effects, which account for heterogeneity between districts. ${z}_{it}^{(\cdot )}$ are exogenous covariates, including time effects; and the ${\beta }^{{(\cdot )}^{T}}$ denotes the coefficient of ${z}_{it}^{(\cdot )}$.

${\omega }_{ji}$ is the spatial contiguity weights matrix, which describes the strength of transmission from region $j$ to region $i$. There are usually three models of neighbourhood weights, including the first-order neighbourhood model, the power law model and the second-order neighbourhood model. The Akaike’s information criterion (AIC) were used to select the model without random effects.The scores rules, such as the logarithmic score (“logs”), the ranked probability score (“rps”), and the Dawid-Sebastiani score (“dss”) were applied to compare the random effect models. These scores measure the predictive quality between the predictive distribution from a fitted model and the observed value, and they give a more robust result for taking into account the random effects. Lower scores correspond to better predictions^22–25^.

### Statistical analysis

Autoregressive integrated moving average (ARIMA) models were applied to predict incidence. The methods above were computed by SAS9.2 (SAS Institute Inc., Cary, NC). The joinpoint regression model was used to examine the trend of the incidence rate of HFRS from 2007 to 2016 by Joinpoint software (version 4.5.0.1). ArcGIS software (version 10.1, ESRI Inc.; Redlands, CA, USA) was used for the spatial autocorrelation analysis. County was adopted as the geographic unit to calculate the spatial autocorrelation. The multivariate time series model was run by R software (version 3.3.1). A *P* value less than 0.05 represented statistical significance for all the tests.

## Result

### Temporal trend, seasonal characteristics and forecast

From January 2007 to December 2016, there were a total of 4,836 HFRS cases and 15 fatal cases reported in Zhejiang Province, China. There were 741, 550, 431, 463, 540, 498, 521, 384, 363 and 345 cases identified in each year of the last decade. Meanwhile, 5, 1, 3, 3, 0, 0, 0, 0, 3 and 0 fatalities occurred each year. This gives 481 average annual cases, 1 average mortality and 0.31% case fatality rate across the observed period.

**Figure 2:**
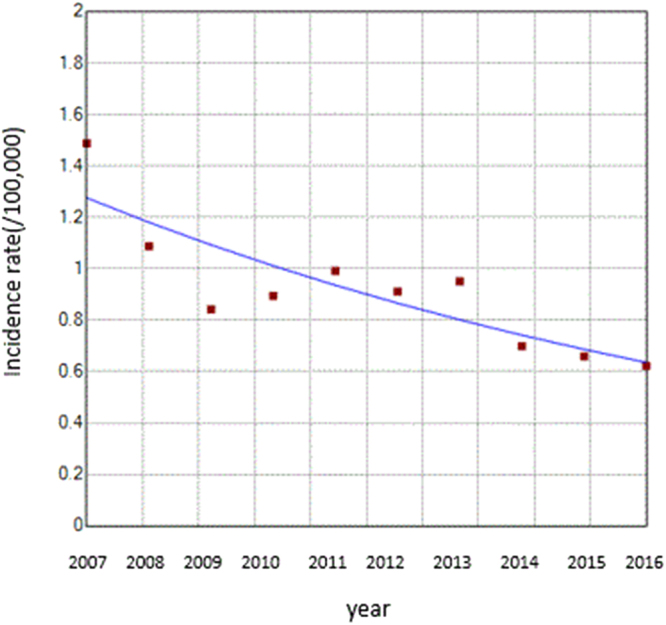
Trend of the incidence rate of HFRS between 2007 and 2016 shown by the joinpoint regression. The red squares denote the observed values of the incidence rates, and the blue line is the slope of the APC. The APC was the annual percent changes.

A fluctuant reduction in the incidence rate of HFRS was seen from 2007 to 2016, with the highest of 1.49 cases per 100,000 in 2007 and the lowest of 0.62 per 100,000 in 2016. The final selected model was the 0 joinpoint relative to the 1 joinpoint (*P* = 0.24). The annual percent change (APC) was −7.40% (95%CI:−10.70 to −4.00) from 2007 to 2016, which is significantly different from zero at the alpha = 0.05 level (test statistic = −4.90, *P* < 0.001), indicating a monotone decreasing trend in the incidence rate (Fig. 2).

**Figure 3:**
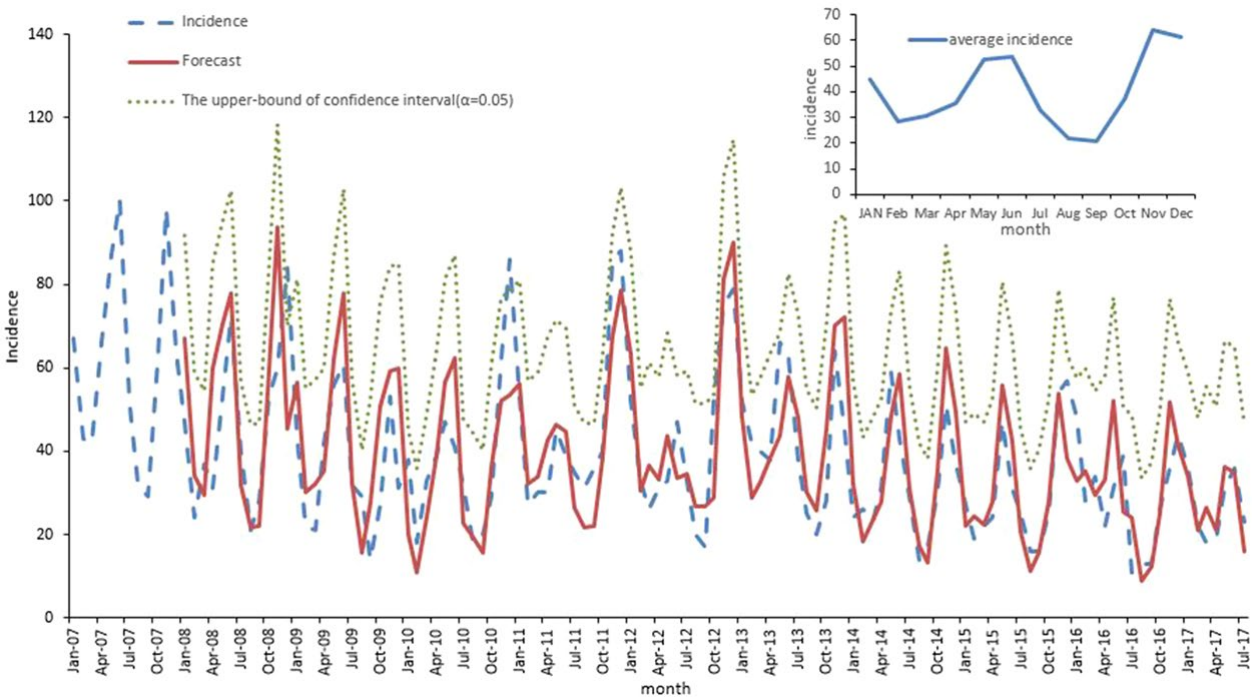
The fitted, predicted, and averaged actual incidence of HFRS in Zhejiang Province, China, from January 2007 to December 2016 at monthly intervals. The green line was the upper-bound predictive value where $\alpha$ was set to be 0.05.

An obvious seasonality was present for the occurrence of HFRS across the ten year period (Fig. 3). There were apparent bimodal curves every year, with two incidence peaks in spring-summer and autumn-winter. The cases reported during May and July accounted for 29.24% of the total cases, whereas they represented 35.26% of the cases reported in November to the next January. The incidence peak in the autumn-winter was slightly higher than that in the spring-summer. Relatively fewer cases were reported in February, August and September compared to the other months.

**Figure 4:**
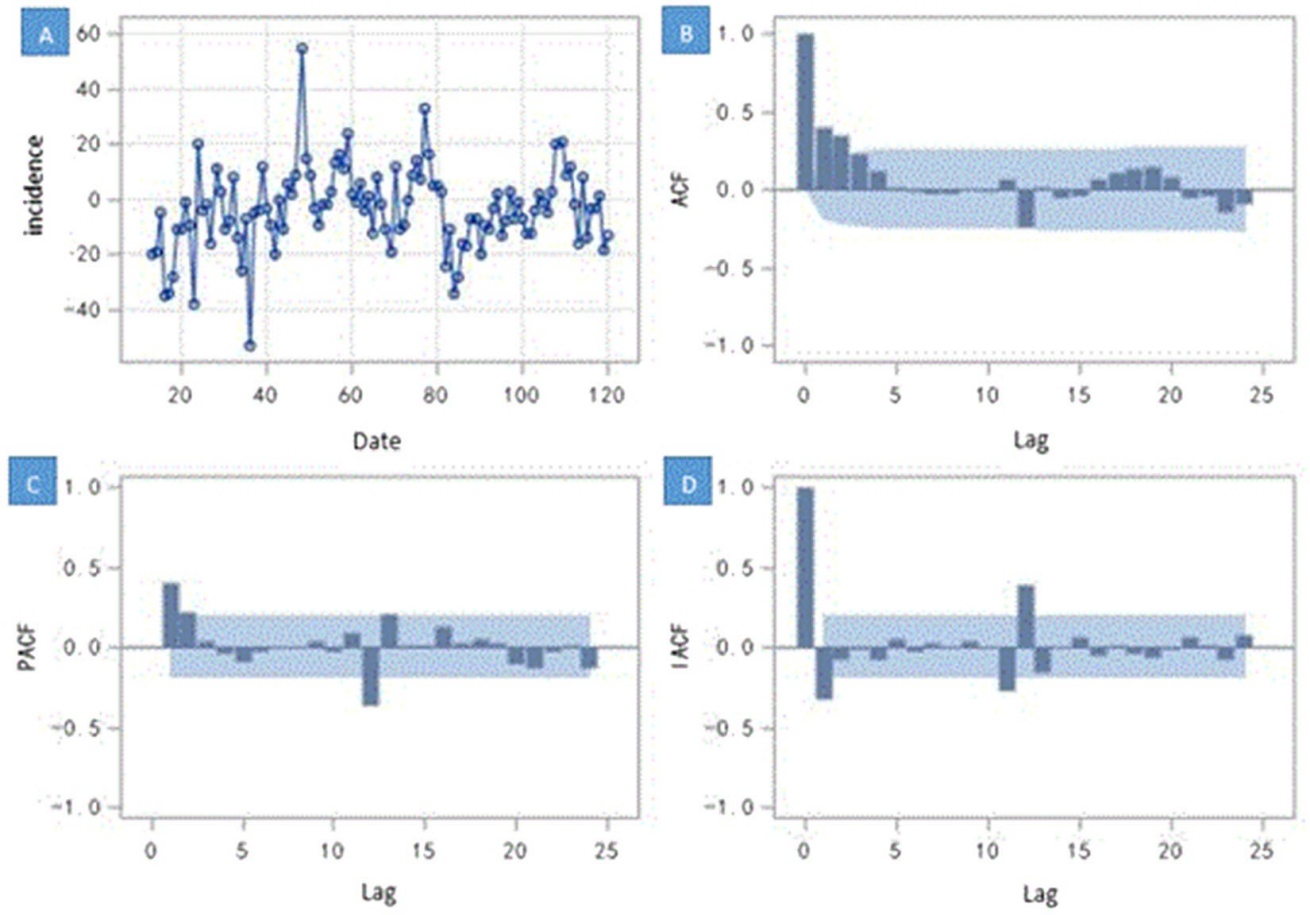
The time series of one step of seasonal difference and its three kinds of autocorrelation function plot. (**A**) The time series after one-step seasonal differences. The x-axis was the time and the y-axis was the difference between the value of incidence and the value at lag 12 months. The plot (**B–D**) show the degree of correlation with past values of the time series. For the plot (**B–D**), the x-axis was the number of periods of the lag, the y-axis was the coefficient of the autocorrelation, partial autocorrelation and inverse autocorrelation, respectively. The blue shadows were the bound of confidence intervals (two times standard deviation) of the coefficient. (**B**) The figure of the autocorrelation of the time series. (**C**) The figure of the partial autocorrelation of the time series. (**D**) The figure of the inverse autocorrelation of the time series.

The monthly incidence data from 2007 to 2016 were used to construct model, and the data from January to July 2017 were used as the test set. According to results of the white noise test (${\chi }^{2}$ = 38.49, df = 6, *P* < 0.0001) in the first step, the null hypothesis of white noise was rejected strongly, which means the time series contains information to model. After checking for the graph of the series, we found that this time series was not stationary for the obvious monthly seasonality. The stationary test shows the null hypothesis could not be rejected for the *P* value was not significant enough (Tau = −2.24, *P* = 0.02), which suggested that the differencing is necessary. We then performed a seasonal differencing (${W}_{t}=(1-{B}^{12}){Y}_{t}$) to ensure that the transformed time series of the monthly HFRS incidence was stationary (Tau = −6.54, *P* < 0.0001) and suitable for constructing the ARIMA model (Fig. 4A). The second step, based on the results of optimal model selection by the Bayesian information criterion (BIC), and the figures of the autocorrelation functions (ACF) and the partial autocorrelation functions (PACF) (Fig. 4B,C), the best model was the ARIMA model (2, 0, 0) × (0, 1, 0)_12_, with a minimum BIC value (5.17).

According to the results of the partial autocorrelation functions (PACF)and inverse autocorrelation function(IACF) (Fig. 4C,D), the partial and inverse autocorrelation coefficients in the lag 12th phase were beyond the 95% confidence interval. Therefore, we further constructed the product model of the ARIMA (2, 0, 0) × (1, 1, 0)_12_ (AIC = 857.82, SBC = 865.87), which was more precise than the model of the ARIMA (2,0,0) × (0,1,0)_12_ (AIC = 871.89, SBC = 879.93). The goodness-of-fit analysis showed that there was no significant autocorrelation between residuals at the 6^th^ lags (${\chi }^{2}$ = 1.06, df = 3,*P* = 0.79), suggesting that the residual series was a white noise series, and the information was fully analyzed. In the third step, the autoregressive parameters were estimated, and the future incidence was forecasted. The autoregressive parameters of the product model were 0.45 and 0.20, and the seasonal autoregressive parameter was −0.41. The predicted data for the actual value of the product model are shown in Table 1. The modelling and the actual time series are presented in Fig. 3. The mean absolute percentage error (MAPE) of the modelling performance and the forecasting performance were 27.53% and 16.29%, respectively. The raw predicted data, the rounding predictive value and the actual data were well matched, and the actual data fell within the predicted 95% confidence interval (Fig. 3).

**Table 1:** Comparison of the predicted HFRS cases and the actual incidence in Zhejiang Province, China.

No. of cases	Jan-17	Feb-17	Mar-17	Apr-17	May-17	Jun-17	Jul-17
Rounding predictive value	34	21	27	21	36	35	16
Raw predictive value	33.97	20.92	26.50	20.96	36.16	34.78	15.99
Actual value	36	22	18	19	31	36	23

### Demographic characteristics of human HFRS cases

Of the total cases, male HFRS human cases predominated, accounting for an average of 73.37% during the ten-year period.We found that the incidence in males was 2.75 times higher than that in females. More than 90% of the cases were in individuals aged 20–75 years. The three age groups with the most reported cases were 40–44 years (13.17%), 45–49 years (13.07%) and 50–54 years (12.66%). Farmers represented the highest proportion (70.45%) of the HFRS cases, followed by workers (13.13%).

### Geographic characteristics and the spatial clustering distribution

**Figure 5:**
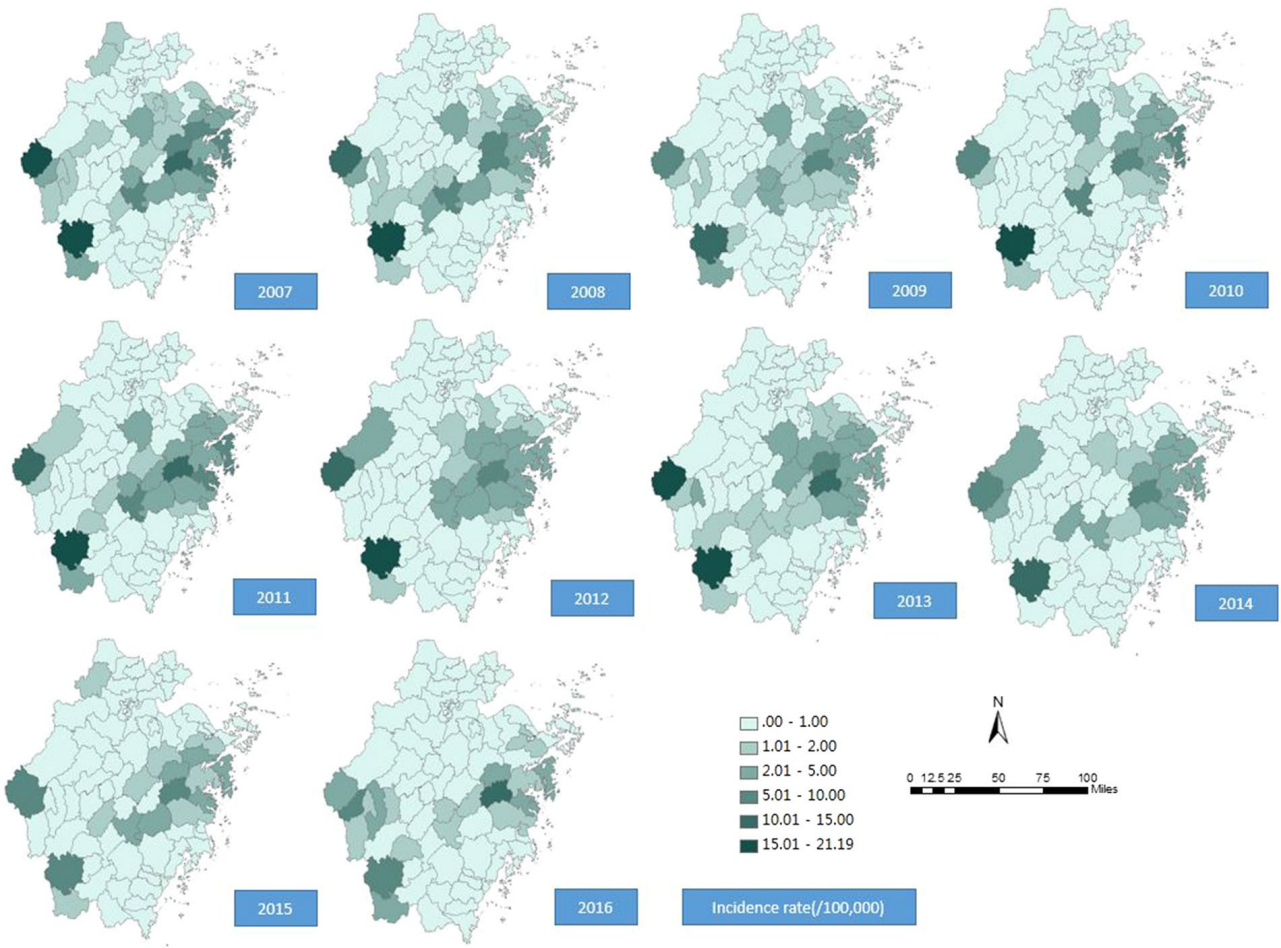
Maps of the incidence rate of HFRS in Zhejiang Province, China, 2007–2016. This map was created by ArcGIS software (version 10.1, ESRI Inc.; Redlands, CA, USA). The homepage of the ArcGIS software was https://www.esri.com/.

Human HFRS cases were reported in most areas of Zhejiang Province, with the exceptions for the following five counties that reported no cases from 2007 to 2016: Jiashan, Haiyan, Deqing, Daishan and Shengsi counties (Fig. 5). The top fourteen counties with average annual incidence rates greater than 2 per 100,000 were Longquan (14.75/10,000), Kaihua (10.61/10,000), Tiantai (9.69/10,000), Xiangshan (4.58/10,000), Xinchang (4.42/10,000), Jinyun (4.36/10,000), Sanmen (3.84/10,000), Fenghua (3.52/10,000), Yinzhou (2.91/10,000), Ninghai (2.84/10,000), Changshan (2.80/10,000), Xianju (2.14/10,000), Linhai (2.11/10,000) and Zhuji counties (2.01/10,000). The counties with high incidence rates were almost all located in the southwest and eastern coastal areas of Zhejiang Province.

According to the global autocorrelation analysis (Table 2), the distribution of the HFRS cases presented a spatial autocorrelation every year from 2007 to 2016. The Moran’s I Index ranged from 0.13 to 0.26 and was highest in 2016 (Index = 0.26, *P* value < 0.001), followed by an index of 0.2085 in 2009.

**Table 2:** The global spatial autocorrelation of HFRS in Zhejiang Province, China, 2007–2016.

Year	Moran’s I Index	Moran’s I Z-score	Moran’s I *P*-value
2007	0.18	2.96	0.003
2008	0.19	3.22	0.001
2009	0.21	3.47	<0.001
2010	0.13	2.17	0.03
2011	0.16	2.63	0.01
2012	0.16	2.75	0.01
2013	0.15	2.62	0.01
2014	0.19	3.16	0.001
2015	0.18	2.92	0.004
2016	0.26	4.25	<0.001

**Figure 6:**
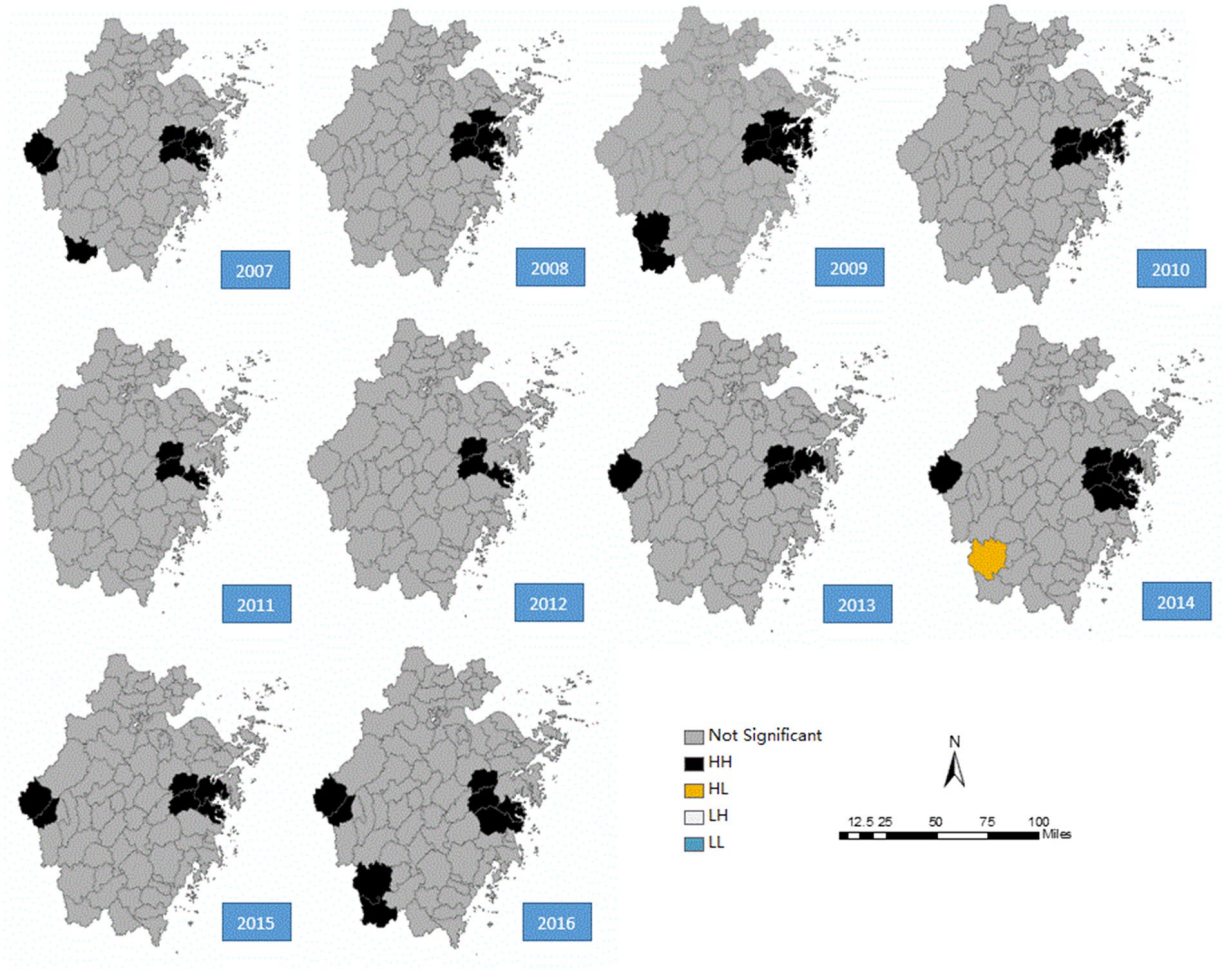
Maps of the local autocorrelation analysis of the incidence rate of HFRS in Zhejiang Province, China, 2007–2016 by the local Moran’s I. This map was created by ArcGIS software (version 10.1, ESRI Inc.; Redlands, CA, USA). The homepage of the ArcGIS software was https://www.esri.com/. The HH was the high-high spatial autocorrelation, the HL was the high-low spatial autocorrelation, the LH was the low-high spatial autocorrelation and the LL was the low-low spatial autocorrelation.

Based on the results of the local Moran’s I autocorrelation (Table 3), there were 54 high-high clusters and 1 high-low cluster in total at the county level during 2007 and 2016, with 7, 5, 8, 4, 3, 3, 4, 7, 6 and 8 clusters each year. It should be noted that Tiantai, Xinchang, Sanmen, Ninghai and Kaihua counties had long-term high-high clusters throughout the ten years, with 10, 10, 8, 7, and 5 high-high clusters, respectively. The hotspots of HFRS transmission were mainly concentrated in eastern areas, including Tiantai, Xinchang, Sanmen and Ninghai counties, which accounted for 63.64% of all the high-high or high-low clusters of Zhejiang Province during the ten years (Fig. 6). Relatively sporadic clusters were observed in the southwest Zhejiang Province. It is interesting that the high-high clusters disappeared in the southwest from 2010 to 2012 and showed a stronger aggregation after 2014. It should also be noted that the counties varied in size, with the largest being 4,452 square kilometres and the smallest being 18.17 square kilometres.

**Table 3:** The local spatial autocorrelation of HFRS in Zhejiang Province, China, 2007–2016.

Year	Area	LMi Index	LMi Z score	LMi *P*-value	Correlation Type	Incidence rate (/100,000)
2007	Xinchang	0.0002	3.75	<0.001	High-High Cluster	6.69
2007	Kaihua	0.0001	3.63	<0.001	High-High Cluster	16.69
2007	Qinyuan	0.00007	3.44	<0.001	High-High Cluster	3.52
2007	Changshan	0.0001	2.23	0.03	High-High Cluster	4.93
2007	Sanmen	0.0001	2.71	0.01	High-High Cluster	6.15
2007	Tiantai	0.0003	4.59	<0.001	High-High Cluster	10.47
2007	Ninghai	0.0002	2.90	0.004	High-High Cluster	4.07
2008	Xinchang	0.0003	5.39	<0.001	High-High Cluster	6.37
2008	Sanmen	0.0001	2.62	0.01	High-High Cluster	4.03
2008	Tiantai	0.0003	5.86	<0.001	High-High Cluster	8.74
2008	Ninghai	0.0003	4.50	<0.001	High-High Cluster	4.35
2008	Fenghua	0.0002	2.11	0.04	High-High Cluster	4.24
2009	Xinchang	0.0003	4.46	<0.001	High-High Cluster	3.26
2009	Qinyuan	0.0001	6.06	<0.001	High-High Cluster	2.43
2009	Longquan	0.0002	6.47	<0.001	High-High Cluster	13.04
2009	Xiangshan	0.0001	3.16	0.002	High-High Cluster	4.24
2009	Sanmen	0.0001	2.68	0.01	High-High Cluster	2.84
2009	Tiantai	0.0004	6.33	<0.001	High-High Cluster	6.41
2009	Ninghai	0.0004	7.01	<0.001	High-High Cluster	3.78
2009	Fenghua	0.0002	2.50	0.01	High-High Cluster	4.58
2010	Xinchang	0.0002	3.23	0.001	High-High Cluster	3.65
2010	Xiangshan	0.0001	2.11	0.04	High-High Cluster	6.37
2010	Tiantai	0.0002	3.97	<0.001	High-High Cluster	7.64
2010	Ninghai	0.0002	3.21	0.001	High-High Cluster	2.71
2011	Xinchang	0.0002	3.05	0.002	High-High Cluster	4.47
2011	Sanmen	0.0002	2.88	0.004	High-High Cluster	5.17
2011	Tiantai	0.0004	6.89	<0.001	High-High Cluster	11.49
2012	Xinchang	0.0002	3.37	<0.001	High-High Cluster	4.18
2012	Sanmen	0.0002	3.28	0.001	High-High Cluster	4.82
2012	Tiantai	0.0004	7.01	<0.001	High-High Cluster	9.89
2013	Xinchang	0.0003	5.82	<0.001	High-High Cluster	6.50
2013	Kaihua	0.0001	2.20	0.03	High-High Cluster	17.95
2013	Tiantai	0.0004	7.18	<0.001	High-High Cluster	11.19
2013	Ninghai	0.0001	2.05	0.04	High-High Cluster	2.77
2014	Xinchang	0.0004	6.28	<0.001	High-High Cluster	3.63
2014	Kaihua	0.0001	2.60	0.01	High-High Cluster	6.94
2014	Linhai	0.0002	2.49	0.01	High-High Cluster	2.87
2014	Longquan	−0.0001	−2.85	0.004	High-Low Cluster	11.85
2014	Sanmen	0.0002	3.94	<0.001	High-High Cluster	2.98
2014	Tiantai	0.0006	11.24	<0.001	High-High Cluster	9.59
2014	Ninghai	0.0003	5.27	<0.001	High-High Cluster	3.07
2015	Xinchang	0.0002	3.07	0.0021	High-High Cluster	2.07
2015	Kaihua	0.0003	8.75	<0.001	High-High Cluster	6.92
2015	Changshan	0.0003	4.72	<0.001	High-High Cluster	5.38
2015	Sanmen	0.0002	3.79	<0.001	High-High Cluster	2.98
2015	Tiantai	0.0004	7.46	<0.001	High-High Cluster	9.32
2015	Ninghai	0.0002	3.27	0.0011	High-High Cluster	1.99
2016	Xinchang	0.0004	6.39	<0.001	High-High Cluster	3.37
2016	Kaihua	0.0002	5.64	<0.001	High-High Cluster	3.65
2016	Linhai	0.0001	2.02	0.04	High-High Cluster	1.82
2016	Qinyuan	0.0001	3.09	0.002	High-High Cluster	2.06
2016	Longquan	0.0001	2.10	0.04	High-High Cluster	7.56
2016	Changshan	0.0004	6.45	<0.001	High-High Cluster	6.60
2016	Sanmen	0.0003	5.53	<0.001	High-High Cluster	3.58
2016	Tiantai	0.0008	14.81	<0.001	High-High Cluster	12.18

### Multivariate time series analysis

The monthly data from 2007 to 2016 were used to construct the multivariate time series model. First, we built two models assuming that the incidence would follow a negative binomial distribution and the Poisson distribution, with the first-order neighbourhood regarded as the neighbourhood weight by default. The AIC of these two models were 15,636.12 and 16,648.44, which suggested that the assumption of the negative binomial distribution of the incidence would be better for modelling. Second, based on the assumption of the negative binomial distribution of the incidence, we built three models including the first-order neighbourhood model, the power law model and the second-order neighbourhood model, and assumed that the neighbourhood weights were one of the three models above. The AIC of the three models were 15,636.12, 15,483.68 and 15,479.48. This shows that the second-order neighbourhood model was slightly better than the other two models. Third, we considered the random effects in the model, and we discovered that the second-order neighbourhood model with random effects introduced by the proper scoring rule was much better than the models without random effects. The final model selected was the second-order neighbourhood model with random effects based on the assessment of the different models (Table 4).

**Table 4:** The comparison of the predictive quality of the random effect models by the scoring rules.

Model	logs	rps	dss
The first-order neighbourhood model	0.86	0.41	0.37
The power law model	0.85	0.41	0.24
The second-order neighbourhood model	0.85	0.41	0.26
The second-order neighbourhood model + random effect	0.73	0.34	−0.07

The estimated random effect variance parameters ${\sigma }_{\lambda }^{2}$, ${\sigma }_{\varphi }^{2}$, and ${\sigma }_{\nu }^{2}$ were 0.33, 1.30 and 2.45, which were used to capture the heterogeneity of HFRS incidence among the different counties. The autoregressive component (${\sigma }_{\lambda }^{2}$) among districts showed little variation. In contrast, considerable spatial variations in the spatio-temporal component (${\sigma }_{\varphi }^{2}$) and the endemic component (${\sigma }_{\nu }^{2}$) were found. This suggested that there was obvious spatial heterogeneity in the endemic component and spatio-temporal component, with little spatial heterogeneity in the autoregressive component.

**Figure 7:**
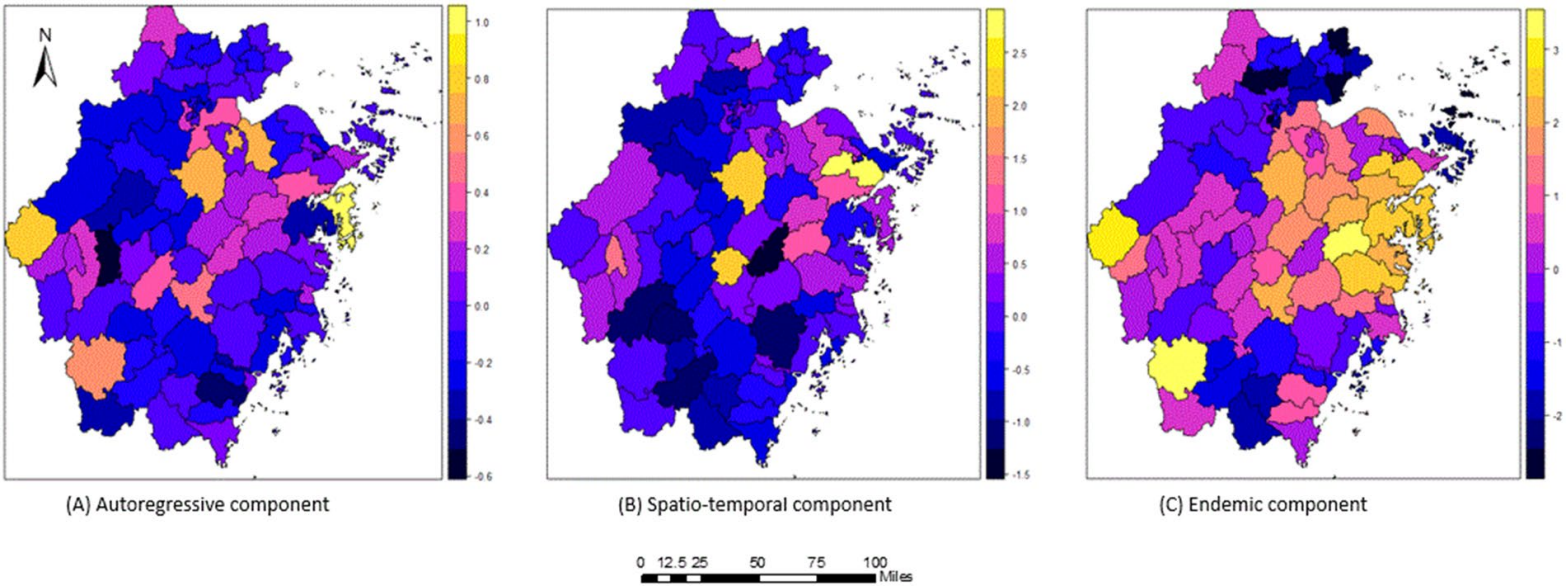
The district-specific random effects of the incidence of HFRS in the multivariate time series model. This map was created by R software (version 3.3.1, http://www.r-project.org/). The colours represented the value of the random effect of the three component at the county level.

For the autoregressive component (Fig. 7A), the random effect values of most counties in Zhejiang Province were lower than 0.4. The top seven counties with random effect values of the autoregressive component greater than 0.4 were Xiangshan (0.96), Kaihua (0.81), Zhuji (0.74), Shangyu (0.72), Yuecheng (0.71), Longquan (0.54) and Jinyun (0.49). The characteristic of spatial dispersion was observed among the counties above. A considerable heterogeneity across counties for the spatio-temporal component is shown in Fig. 7B. The counties with high values of the random effect of the spatio-temporal component were Yinzhou county (2.64), Zhuji county (2.32), Yongkang county (2.25), Kecheng county (1.31), Tiantai county (1.12) and Fenghua county (1.02). The districts with higher values of the random effect of the spatio-temporal component were mainly located in the eastern parts of Zhejiang Province. Similarly, there was significant spatial heterogeneity among the districts for the endemic component (Fig. 7C). Moreover, the counties with a high value of the random effect of the endemic component were almost all located in the middle eastern parts of the province. It should be noted that Kaihua and Longquan counties had high random effect values and were located in southwest Zhejiang Province. Further, the top 12 counties with high random effect values of the endemic component were Tiantai county (3.16), Longquan county (3.15), Kaihua county (2.79), Linhai county (2.59), Yinzhou county (2.47), Xiangshan county (2.45), Ninghai county (2.40), Jinyun county (2.30), Xinchang county (2.14), Zhuji county (2.11), Sanmen county (2.04) and Fenghua county (2.04) (Table 5).

**Table 5:** The top ten counties with the highest random effects of the incidence of HFRS.

county	autoregressive component	county	spatio-temporal component	county	endemic component
Xiangshan	0.96	Yinzhou	2.64	Tiantai	3.16
Kaihua	0.81	Zhuji	2.32	Longquan	3.15
Zhuji	0.74	Yongkang	2.25	Kaihua	2.79
Shangyu	0.72	Kecheng	1.31	Linhai	2.59
Yuecheng	0.71	Tiantai	1.12	Yinzhou	2.47
Longquan	0.54	Fenghua	1.02	Xiangshan	2.45
Jinyun	0.49	Nanxun	0.92	Ninghai	2.40
Wuyi	0.34	Xinchang	0.86	Jinyun	2.30
Xiaoshan	0.34	Haishu	0.81	Xinchang	2.14
Fenghua	0.33	Yuyao	0.75	Zhuji	2.11

**Figure 8:**
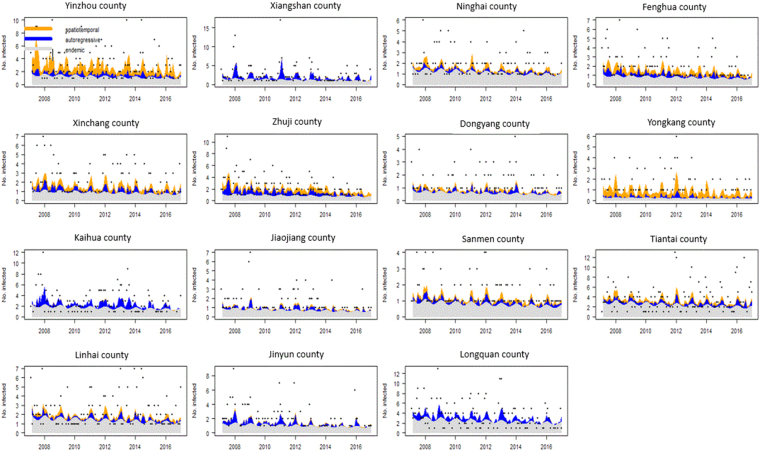
Fitted components in the multivariate time series model for the 15 counties with more than 100 cases during the past ten years. The black dots represent the monthly counts of incidence, the light grey area shows the endemic component, the blue area shows the autoregressive component, and the yellow area corresponds to the spatio-temporal component.

To judge the relative importance of the three model components in the high incidence areas (>100 cases over ten years), we plotted the mean components along with the observed counts (Fig. 8). The relative contributions of the three components in driving the prevalence of HFRS over time were exhibited by these figures(Fig. 8), and the seasonal characteristics were displayed at the same time.

There were considerable differences in the multivariate time series models between the aforementioned high incidence areas. To a large degree, most of the high incidence areas, except for Yongkang county, were affected by the endemic component for the entire ten year period. Clear seasonality with two peaks was observed every year. Yongkang county, unlike other counties, was mainly affected by the spatio-temporal component. Yinzhou county was influenced equally by the spatio-temporal and the endemic components. Unlike Yongkang and Yinzhou counties, the other 8 counties were all closely located in the middle eastern portion of Zhejiang Province and were mainly affected by the endemic component, while they were slightly influenced by the spatio-temporal and the autoregressive factors. These counties were Ninghai, Fenghua, Xinchang, Zhuji, Dongyang, Sanmen, Tiantai and Linhai. It is interesting that five counties (Xiangshan, Jiaojiang, Kaihua, Jinyun and Longquan) dispersedly located in the border areas of Zhejiang Province were the most influenced by the autoregressive factor instead of the endemic component. The autoregressive influence was clearly observed in the incidence peak and exhibited a decreasing trend, especially in the recent years.

## Discussion

According to our research, there were more than 300 HFRS cases identified every year during the last ten years, some of which led to deaths. The epidemic of HFRS remainsa public health threat in Zhejiang Province, China. This province is one of the top six provinces with the highest incidence rate of HFRS^4^, though its deaths and fatality rates are much lower than the overall averages for China. The reason for the high incidence rate but low fatality rate may be that the overall diagnostic ability and medical treatment level in Zhejiang Province is preferable compared to the other provinces with high incidence. There fore, those infected cases can be identified early and treated over time. Based on the results of the joinpoint regression analysis, we found an obvious decreasing trend in the incidence rate from 2007 to 2016. One of the reasons for the decline is the high urbanization rate and GDP (Gross Domestic Product) per capita Zhejiang Province (ranked fifth in China with 12,635 dollars in 2016). Many studies found that the HFRS incidence decreased with an increase in per capita GDP and urbanization rate, since economic development may reduce HFRS transmission by decreasing rodent density^26^ and thus the risk of excreta exposure^27^. Another reason may be because Zhejiang Province has been one of the trial areas for the implementation of the National Expanded Programme on Immunization with the hantavirus vaccine since 2008^4^.

Consistent with previous research^4,8^, there were two peaks of incidence in Zhejiang Province,with close numbers of cases in these two peaks. As we know, previous studies^4^ suggest that HFRS in China is mainly caused by two types of hantaviruses including HTNV transmitted by *Apodemus agrarius* and SEOV transmitted by *Rattus norvegicus*. In addition, the rodent population density determines the incidence in humans^1^. We observed two peaks (June to July and November to January in the next year) for the incidence curve of Apodemus-type HFRS and only one peak (March to May) for the epidemic of Rattus-type HFRS. Thus, the epidemics in Zhejiang Province may have been caused by both *Apodemus agrarius* and *Rattus norvegicus*, contributing to the seasonality of the two peaks. Based on the seasonality and the declining trend in the incidence, the monthly incidence is meaningful in the temporal dimension against the assumption of white noise and is stationary after one-step seasonal differences. It is suggested that this time series is suitable for constructing the ARIMA model, which is widely used to assess and forecast the incidence of infections^13–16^. One important conclusion from our model is that the incidence of HFRS in Zhejiang Province among the next six months will be similar to that in the same period from 2016 and maintain the seasonal characteristics. It should be noted that the performance of our model is similar to other research models^17,28^. It is worth mentioning that the prediction of the time series by the ARIMA model is only based on its past value. Therefore, the epidemiological factors driven the transmission of the disease should be kept the similar trend and seasonality to ensure an accurate forecast.

Consistent with previous research^29,30^, middle-aged male HFRS cases predominated, and farmers accounted for the highest proportion of all occupations. To our knowledge, the hantavirus is most likely transmitted to humans through the inhalation of aerosolized excreta from infected rodents^1^. The variations in the distribution of gender, age and occupation are most likely caused by varying chances of exposure to the infected rodents and their excreta^29^. Middle-aged male farmers are expected to participate more frequently in outdoor activities, such as harvesting, which would increase exposure to infected rodents.

According to the incidence map and the Moran’s I autocorrelation during the last decade, significant differences in the incidence and regional distribution were identified, and there were obvious clusters in some counties of Zhejiang Province. Most of the long-term hotspots were found in eastern areas, and some short-term high clusters were observed in the southwest. The finding suggests that the incidences of HFRS were similarly high among these areas Hence, more attention is needed to prevent epidemics in these areas. An interesting observation was that the high clusters disappeared in the southwest from 2010 to 2012 but reoccurred and expanded from 2013. The possible explanation is the selection of the hotspots. When we conducted the local autocorrelation analysis to detect the spatial similarity, the areas with the similar high values of the incidence rates were identified as the hotspots. We found that the incidence rates in Longquan county and Kaihua county were consistently high during the 10-year period compared to their neighboring areas with relatively low and varying incident rate. Nevertheless, it is worth noting that the epidemic in Kaihua county and Longquan county may expand to neighboring areas and act as reservoirs for the virus.

To our knowledge, the spatio-temporal heterogeneity of the incidence was affected by various factors such as temperature, precipitation, humidity, NDVI (normalized difference vegetation index), land use, rodent population density and human activity^1,26,31^. Different levels of seriousness of the risk factors can lead to different patterns of disease transmission, which may have caused the variations in the distribution of the epidemics among the counties. Therefore, a multivariate time series model with random effects accounting for the effect produced by unobserved factors^25^ was constructed to analyze the spatial and temporal occurrence of HFRS and to capture the heterogeneity in the component driving HFRS transmission. Based on the model, obvious spatial heterogeneity in the endemic and spatiotemporal components were identified. The high value of the endemic component was found in many counties, especially those located in the middle eastern areas of Zhejiang Province. Except for Yongkang county, all the other counties with a high incidence were mainly affected by the endemic component, which suggests that most infections in these areas can be explained by climatological and ecological changes, socioeconomic activities, living conditions and rodent density^1,26^. Moreover, considerable seasonality was observed in the time series of components, which shows that seasonal changes in climatological and ecological factors, such as increased rainfall, warmer winters, and abundant rodent food sources, will lead to an increase in the rodent population and contribute to the peaks in HFRS cases several months later^26^. In addition, the spatio-temporal and the autoregressive components also affected the disease transmission. Yongkang and Yinzhou counties, which are located near the eastern high-high cluster areas, were obviously affected by the spatio-temporal component, which indicates that the cases in these areas may have been imported from neighbouring areas with high incidence. For example, individuals who work in neighbouring areas with high incidence but live and seek medical advice in their hometowns were affected. Furthermore, we found that the counties Xiangshan, Jiaojiang, Kaihua, Jinyun and Longquan, which were geographically separated from each otherwere influenced by the autoregressive factors instead of the endemic components. In these counties, the epidemic effect in the previous season continued and partly contributed to the later peaks. Eight counties (Ninghai, Fenghua, Xinchang, Zhuji, Dongyang, Sanmen, Tiantai and Linhai) closely located in the middle eastern area of Zhejiang Province had the most high-high clusters, and the spatio-temporal and the autoregressive component contributed to rest of the epidemics. This result suggests that the cross-regional importation of HFRS, previous epidemics, and climatological and ecological factors altogether caused the disease and led to long-term clusters.

Based on the heterogeneity of the components driving HFRS transmission, targeted preventive efforts are needed in the different areas. For most counties with a high incidence influenced mainly by the endemic component, coordinated rodent-control efforts could be the most appropriate way to prevent human HFRS^1^. Another important measure is educating the public, especially farmers and forest workers, to improve general hygiene. Means such asusing face masks when working in agriculture should be adopted to decrease the risk of inhaling virus-carrying particulates. For endemic areas, the HFRS vaccine may have a considerable effect on HFRS prevention and can be used as aneffective resolution for HFRS control in the future^32^. For Yongkang and Yinzhou county, where were obviously affected by the spatio-temporal component instead of endemic factors and epidemic control, it is recommended to use rodent control in neighbouring areas such as Ninghai, Fenghua, Dongyang and Tiantai counties in order to decrease incidence. For Xiangshan, Jiaojiang, Kaihua, Jinyun and Longquancounty, where the epidemic was mainly effected by the autoregressive factor instead of the endemic component, early implementation several months ahead of the peak may effectively decrease the number of cases of HFRS in the peak season.

Several limitations should be noted within our study. First, the data regarding the risk factors including climatic factors, pathogen and host dynamics, socioeconomic status and human activities were not collected. Consequently, these factors could not be compared and incorporated into the multivariate time series model. The correlation between these risk factors and the incidence of HFRS was also not studied. In order to achieve an accurate forecast of the HFRS incidences, future studies should incorporate these cofactors in the multivariate time series model and further compare the model with others including artificial neural network, Markov model and other models.

Second, our sample included the cases of HFRS reported from a passive surveillance system. Future studies should consider the reporting level, e.g., including the mild and subclinical cases that did not seek medical care. Third, the relatively low diagnostic levels in some counties also led to the underestimation of incidence. It is necessary to take into account the diagnostic to correct the incidence for future research.

In conclusion, Zhejiang Province is a province with high incidence of HFRS in China. However, a significant decreasing trend in the incidence rate was seen from 2007 to 2016, according to the joinpoint regression analysis. The distribution of the seasonal peak, gender, age and occupation was similar to previous studies. Obvious clusters were identified in the eastern and southwestern areas of Zhejiang Province. A spatial heterogeneity in the component driving the transmission of HFRS was identified from the multivariate time series model. This suggests that targeted preventive efforts should be made in different areas based on the main component contributing to the epidemics. Case surveillance, especially for mild cases, should strengthen ongoing research with the incorporation of risk factors.
